# Shared Odds of *Borrelia* and Rabies Virus Exposure in Serbia

**DOI:** 10.3390/pathogens10040399

**Published:** 2021-03-28

**Authors:** Pavle Banović, Adrian Alberto Díaz-Sánchez, Dragana Mijatović, Dragana Vujin, Zsolt Horváth, Nenad Vranješ, Zorana Budakov-Obradović, Nevenka Bujandrić, Jasmina Grujić, Abdul Ghafar, Abdul Jabbar, Verica Simin, Dasiel Obregón, Alejandro Cabezas-Cruz

**Affiliations:** 1 Ambulance for Lyme Borreliosis and Other Tick-Borne Diseases, Department of Prevention of Rabies and Other Infectious Diseases, Pasteur Institute Novi Sad, 21000 Novi Sad, Serbia; Draganav77@gmail.com; 2 Department of Microbiology with Parasitology and Immunology, Faculty of Medicine in Novi Sad, University of Novi Sad, 21000 Novi Sad, Serbia; 3 Department of Biology, University of Saskatchewan, 112 Science Place, Saskatoon, SK S7N 5E2, Canada; adiasanz88@gmail.com; 4 National Reference Laboratory for Rabies, Department of Microbiology, Pasteur Institute Novi Sad, 21000 Novi Sad, Serbia; favn.paster@gmail.com; 5 Agricultural School, Maršala Tita 167, 24300 Bačka Topola, Serbia; cibi@stcable.net (Z.H.); Luketic.s@mts.rs (V.S.); 6 Department for Research & Monitoring of Rabies & Other Zoonoses, Pasteur Institute Novi Sad, 21000 Novi Sad, Serbia; nenad.vranjes@gmail.com; 7 Faculty of Medicine in Novi Sad, University of Novi Sad, 21000 Novi Sad, Serbia; zorana.budakov-obradovic@mf.uns.ac.rs (Z.B.-O.); nevenka.bujandric@mf.uns.ac.rs (N.B.); jasmina.grujic@mf.uns.ac.rs (J.G.); 8 Blood Transfusion Institute Vojvodina, 21000 Novi Sad, Serbia; 9 Department of Veterinary Biosciences, Melbourne Veterinary School, Faculty of Veterinary and Agricultural Sciences, The University of Melbourne, Werribee, VIC 3030, Australia; aghafar@student.unimelb.edu.au (A.G.); jabbara@unimelb.edu.au (A.J.); 10 School of Environmental Sciences, University of Guelph, Guelph, ON N1G 2W1, Canada; dasieloa@uoguelph.ca; 11 Center for Nuclear Energy in Agriculture, University of São Paulo, Piracicaba, São Paulo 13400-970, Brazil; 12 Anses, INRAE, Ecole Nationale Vétérinaire d’Alfort, UMR BIPAR, Laboratoire de Santé Animale, F-94700 Maisons-Alfort, France

**Keywords:** rabies, *Borrelia*, seroprevalence, exposure, relative odds

## Abstract

Lyme borreliosis (LB) is the most common tick-borne disease in Serbia and other European countries. Rabies is a fatal zoonosis distributed worldwide and is caused by the rabies virus. Professionals at risk of rabies—including veterinarians, hunters, communal service workers, and forestry workers—overlap with some professions at a higher risk of exposure to tick bites and tick-borne pathogen infections. We hypothesized that individuals identified by the public health system as at risk of rabies virus infection, and consequently vaccinated against rabies virus, also share a higher likelihood of *Borrelia* exposure. To test our hypothesis, a case-control study was carried out during 2019 in Serbia to determine the seroprevalence of anti-*Borrelia* antibodies in two case groups (individuals at risk and vaccinated against rabies virus) and a control group (individuals without risk of rabies). Individuals vaccinated against rabies following either “pre-exposure protocol” (PrEP, *n* = 58) or “post-exposure protocol” (PEP, *n* = 42) were considered as rabies risk groups and healthy blood donors (*n* = 30) as the control group. The results showed higher *Borrelia* seroprevalence in PrEP (17.2%; 10/58) and PEP (19.0%; 8/42) groups compared with the control group (6.67%; 2/30). Furthermore, odds ratio (OR) analysis showed that risk of rabies (in either the PrEP (OR = 2.91) or PEP (OR = 3.29) groups) is associated with increased odds of being seropositive to *Borrelia*. However, the difference in *Borrelia* seroprevalence between groups was not statistically significant (Chi-square (χ²) test *p* > 0.05). The shared odds of LB and rabies exposure found in this study suggest that, in countries where both diseases occur, the common citizen can be at risk of both diseases when in a risky habitat. These findings are important to guide physicians in targeting high-risk groups, and diagnose LB, and to guide decision-makers in targeting control and prevention measures for both infections in risk areas.

## 1. Introduction

Ecological perturbations due to land use by humans, growing human population, globalization, and climate change are associated with an increased incidence and prevalence of vector-borne diseases. Higher average temperatures associated with climate change are suitable for most arthropod vectors, which may explain the territorial expansion and increased abundance of different vectors. Ticks are the main vectors for animal and human diseases in Europe, and their geographic ranges have expanded in recent years [1,2,3]. *Ixodes ricinus* (Genus: *Ixodes*, Family: Ixodidae), the most important tick vector of human diseases in Europe, is a generalist tick that can feed on several animal species, including humans—who are considered accidental hosts [4,5]. *Ixodes ricinus* is mostly found in deciduous forests where small mammals and deer serve as its main hosts. However, this tick species can also be found in swamps and meadows during periods of high rainfalls [6,7]. Diseases transmitted by *I. ricinus* include tick-borne encephalitis, human granulocytic anaplasmosis and other rickettsial diseases, babesiosis and Lyme borreliosis (LB) [8,9]. *Borrelia* infection in Europe is caused by the members of the *Borrelia burgdorferi* sensu lato (s.l.) complex, including *Borrelia burgdorferi* sensu stricto (s.s.), *Borrelia afzelii*, and *Borrelia garinii*, and the estimated incidence of LB in the region ranges from 85,000 to more than 200,000 cases per year [10,11]. Although less frequent, LB can also be caused by *Borrelia spielmanii* and some pathogenic strains of *Borrelia lusitaniae*, *Borrelia valaisiana*, and *Borrelia bissettii* [11,12]. The spirochaete are transmitted enzootically between ticks and their hosts. In Serbia, as in other countries of Europe, *I. ricinus* is responsible for the transmission of several tick-borne diseases (TBDs) including LB, tick-borne encephalitis, and rickettsioses [13,14,15,16]. Among these TBDs, LB is the most common in Serbia [13,17], where *Borrelia* DNA have been detected in *I. ricinus* collected from several regions [18,19], and about 50% of the tested ticks were found positive for at least one member of *B. burgdorferi* s.l. complex [18]. Due to the absence of a national guideline for diagnostics and treatment of TBDs, LB is possibly an underestimated cause of disease in Serbia.

Rabies is a lethal zoonosis with worldwide distribution and is caused by rabies virus (Genus: *Lyssavirus*, Family: Rhabdoviridae), which affects the central nervous system of mammals and leads to signs of encephalomyelitis with almost 100% fatality rate [20]. The virus normally circulates within its natural hosts (i.e., species of the orders Carnivora and Chiroptera). Hosts, such as humans, who are not reservoirs for rabies virus, get infected as a consequence of spillover events when an infected natural host (e.g., fox, dog, bat) transmit the virus via licking, scratching, or biting [21]. The number of human deaths globally due to dog-associated rabies is estimated to be 59,000 annually, and most of the victims are children under 15 years [21]. In Serbia, dog-associated rabies was a public health problem until 1980s. Since then, most rabies cases in Serbia have been associated with foxes, and thus this animal has become the main reservoir species for rabies virus in the country [22]. In humans, without preventive vaccination or earlier post-exposure vaccination/treatment, the disease is fatal. Accordingly, “pre-exposure protocol” (PrEP) immunization against rabies is recommended for professionals (such as veterinarians, hunters, communal service workers, and forestry workers) in close contact with wild and stray animals, and “post-exposure protocol” (PEP) immunization is recommended for individuals who were in contact with a rabid or suspected-rabid animal, independent of profession-associated risks [23]. Annually, about 700 people receive anti-rabies prophylaxis in Serbia, from which 50% are indications of PEP. Rabies is a notifiable disease in Serbia, and except for a case of fox-transmitted rabies detected in 2018 (unpublished data), the effective vaccination strategy has controlled the occurrence of human rabies cases in Serbia [24]. Due to their professional activities and/or lifestyle, risk groups included in the PrEP and PEP vaccination protocols could also be at high risk of exposure to tick bites and LB. 

Considering the above evidence, we hypothesized that individuals identified by the public health system as at risk of rabies virus infection also share a high likelihood of being bitten by *I. ricinus* during field activities and, in consequence, to be more exposed to *Borrelia* infection than individuals with no regular access to risk areas. To test our hypothesis (i.e., the odds of *Borrelia* seropositivity are higher among individuals at risk of rabies virus exposure than among individuals at no risk of rabies virus exposure), we designed a case-control study to compare the seroprevalence of anti-*Borrelia* antibodies in individuals immunized against rabies (two case groups) and healthy blood donors with no profession-associated accesses to risk areas (control group) in Serbia and Bosnia and Herzegovina. Such valuable information would be highly useful for the planning of prevention and control measures, particularly for personnel at high risk of LB.

## 2. Materials and Methods

### 2.1. Ethical Statement

This study was approved by the Novi Sad Ethical Committee at the Faculty of Medicine, the University of Novi Sad (approval number 01-39/266/1), and was conducted according to the Declaration of Helsinki and The Patient Rights Law of the Republic of Serbia. Written informed consents were obtained from blood donors to allow the use of their blood samples for this study.

### 2.2. Study Design and Sampling Strategy

An observational, case-control study was conducted during the year 2019 in Serbia and Bosnia and Herzegovina to compare the seroprevalence of anti-*Borrelia* IgG among individuals at risk, or not, of rabies virus exposure. Accordingly, two groups of individuals were considered at risk of rabies virus exposure. The first group included individuals who live, or spend time, in rural areas or forests and after being bitten by rabid or rabies-suspected animals, were vaccinated against rabies and treated with anti-rabies sera (hereafter referred to as the “post-exposure protocol” group, PEP). The second group included professionals (i.e., veterinarians, community service workers, foresters) who had received preventive rabies immunization (hereafter referred as the “pre-exposure protocol” group, PrEP) due to profession-associated increased risk of rabies virus exposure. Sera from clinically healthy blood donors without an identified profession-associated risk of rabies virus exposure were selected as the control group. Anti-*Borrelia* antibodies were tested in all sera samples by indirect immunofluorescent test (IFAT). The association between the seroprevalence of *Borrelia* spp. and rabies virus exposure was explored in potential risk groups, including age (children and teenagers: 0–19; adults: 20–59; and seniors: >60 years), gender (male and female), residence (North, South, and Central Serbia and Bosnia and Herzegovina (Sarajevo)) and the anti-rabies immunization protocol (PEP and PrEP). Socio-demographic data were obtained through medical documentation available at the Pasteur Institute Novi Sad (PEP and PrEP groups) and Blood Transfusion Institute of Vojvodina (control group).

### 2.3. Selection of Samples in PEP and PrEP Groups

For this study, sera samples of PEP (*n* = 42, Supplementary Materials Table S1) and PrEP (*n* = 58, Supplementary Materials Table S2) individuals were randomly selected from the sera bank at the National Reference Laboratory for Rabies (NRLR), Pasteur Institute Novi Sad, Serbia. The NRLR is a national-level institution that archives sera samples from individuals immunized against rabies at local “anti-rabies stations” scattered across the territory of Serbia. All individuals in the PEP and PrEP groups were immunized against rabies with the vaccine Verorab^®^ (Sanofi Pasteur, Paris, France). In the case of PEP, along with the first vaccine dose, human rabies immune globulin (HRIG) (Blood Transfusion Institute of Serbia, Belgrade) was administered to each patient as passive immunization. Accordingly, all the sera samples in the PEP and PrEP groups contained neutralizing anti-rabies antibodies in titer 0.5 ≤ IU detected with the golden standard assay i.e., rapid fluorescent foci inhibition test (RFFIT). Sera were stored at −80 °C for at least one year after the detection of rabies-neutralizing antibodies titer.

### 2.4. Selection of Healthy Blood Donor Samples

Healthy blood donor sera samples (*n* = 30, Supplementary Materials Table S3) used as the control group were acquired from the Blood Transfusion Institute of Vojvodina, Serbia. None of the individuals in this group had professions associated with risk of exposure to rabies virus (Supplementary Materials Table S3). As part of the routine screening of blood donors by the Blood Transfusion Institute of Vojvodina, control samples were analyzed for and tested negative for the presence of antibodies against *Treponema pallidum* and antigen, as well as for antibodies and the DNA of the human immunodeficiency and hepatitis C viruses.

### 2.5. Borrelia Culture and IFAT Antigen Preparation

*Borrelia* spirochetes were grown in complete BSK-H medium (Sigma-Aldrich, Saint Louis, MO, USA, Cat. B8291) at 33 °C [25]. The culture (2 mL) was pelleted down by centrifugation at 1200 × g for 15 min and washed twice in sterile phosphate buffered saline (PBS; pH 7.4). The density of *Borrelia* culture was adjusted to approximately 3 × 10^7^ organisms/mL. We used the whole spirochetes as antigen and 0.2 mL of the suspension of *Borrelia afzelii* in PBS was added to microscopic slide coated with poly-L-lysine (Sigma-Aldrich, Saint Louis, MO, USA, Cat. P0425) and left to dry at room temperature. Antigenic fields were then encircled with hydrophobic PAP pen (Abcam, Cambridge, United Kingdom, Cat. ab2601). The *B. afzelii* strain, part of a *Borrelia* spp. collection, was kindly donated by the Group of Medical Entomology, Institute for Medical Research, University of Belgrade.

### 2.6. Detection of Anti-Borrelia Antibodies by IFAT

Anti-*Borrelia* IgG were detected by an in-house IFAT using *B. afzelii* antigens on microscopic slides. Previous to the analysis of the samples of the study, the in-house IFAT assay was validated using Euroimmun commercial IFAT assay (FI 2131-1 G, Euroimmun, Lubeck, Germany) as a reference and according to the guidelines and methodology for validation of diagnostic tests described in the *Manual of Diagnostic Tests and Vaccines for Terrestrial Animals of the World Organization for Animal Health* (OIE) [26]. Optimal antigen concentration as well as sera and antibody dilutions were defined using titration assays, where PBS (pH 7.4) was used for dilution of examined sera. Test sera were diluted to 1:100 and incubated with antigen-coated slides for 45 min in a humidified chamber at 37 °C. Following two PBS washes, goat anti-human IgG labelled with the green fluorescent dye CF™488A (Sigma-Aldrich, Saint Louis, MO, USA, Cat. SAB4600041) was added as a secondary antibody at 1:20 working dilution (antibody was diluted in PBS and Evans blue was added in final mixture to achieve counterstain concentration of 0.5%) and incubated for 45 min in humidified chamber at 37 °C. The slide was then washed twice with PBS (pH 7.2, Thermo Scientific, Waltham, MA, USA). Presence of *B. afzelii* antigens on the slides was confirmed by adding to each slide a positive control (i.e., *B. afzelii* antigen recognition by polyclonal anti-*Borrelia* antibodies diluted 1:20 and labeled with fluorescein isothiocyanate (FITC) (Invitrogen, Carlsbad, CA, USA, Cat. PA1-73005). The absence of non-specific reactions of the secondary antibody was confirmed by adding a negative control (i.e., *B. afzelii* antigen directly exposed to secondary antibodies without primary antibodies) on each slide.

Fluorescence reactions were visualized on a Leica DM 3000 microscope (Leica, Wetzlar, Germany) with an excitation wavelength of 515–560 nm. Results were considered positive when an intense fluorescence reaction was detected at 1:100 or higher sera dilution. In case of weak fluorescence at 1:100 dilution, the sample was declared as borderline and additionally tested using the second-tier *recom*Blot test (Mikrogen Diagnostik, Neuried, Germany, Cat. 4272), based on recombinant *Borrelia* spp. antigens. Inconclusive results in the second-tier test were considered negative. 

### 2.7. Data Analysis

The association between the seroprevalence of anti-*Borrelia* IgG and the exposure to rabies virus between groups of cases (PEP and PrEP) and control (healthy donors), and further between demographic groups (i.e., gender, age, residence, and immunization protocol), was explored on a series of contingency tables (i.e., observed and expected events for each group), assessed using two-tailed Chi-square (χ²) test; Yates’s correction was used to prevent overestimation of statistical significance because of the small sample size (*n*) in the study. The test was implemented in the statistical software package IBM SPSS Statistics 25 (IBM, Armonk, NY, USA). The association between *Borrelia* spp. seropositivity and rabies virus risk exposure in the case groups (i.e., PEP and PrEP) and control group (i.e., healthy donors) were calculated by the odds ratio (OR) (CI 95%). Statistical significance was considered for *p*-values < 0.05. Data analysis was performed using GraphPad Prism v.8.0.1 (GraphPad Software Inc., La Jolla, CA, USA).

## 3. Results

### 3.1. Association between Borrelia Seroprevalence and Risk of Rabies Exposure 

Anti-*Borrelia* IgG were detected in 20 of the 130 analyzed samples (15.38%; CI, 9.10–21.66). In a second-tier test, three samples (2 PEP and 1 PrEP) were borderline and considered as negative. The highest *Borrelia* spp. seroprevalence (18/100, 18%) was found among individuals at risk of rabies virus exposure (i.e., PEP and PrEP case groups). Specifically, anti-*Borrelia* IgG were detected in 19.0% (8/42) and 17.2% (10/58) samples of the PEP and PrEP groups, respectively. In contrast, only 6.67% (2/30) samples were seropositive for *Borrelia* spp. in the healthy donors group (i.e., control group), composed by individuals without risk of rabies virus exposure (Table 1).

*Borrelia* spp. seroprevalence in PEP (χ² = 2.24, *p* = 0.13) and PrEP (χ² = 1.87, *p* = 0.17) case groups was not statistically significant compared with the healthy donors control group. In addition, no significant difference (χ² = 0.05, *p* = 0.81) was found in the *Borrelia* spp. seroprevalence of the case groups. Although the differences did not reach statistical significance, odds ratio (OR) analysis showed that *Borrelia* spp. seroprevalence was positively correlated (OR > 1) with risk of rabies virus exposure in the PEP (OR = 3.29; CI, 0.65–16.78; *p* = 0.13) and PrEP (OR = 2.91; CI, 0.59–14.27; *p* = 0.17) case groups compared with the healthy donors control group. In contrast, comparison of *Borrelia* spp. seroprevalence in PEP and PrEP case groups revealed an OR very close to 1 (OR = 1.12; CI, 0.40–3.15; *p* = 0.13), suggesting that the outcome (i.e., *Borrelia* spp. seroprevalence) is independent of the vaccination protocol (i.e., PEP and PrEP).

### 3.2. Association between Regional Distribution of Borrelia spp. Seroprevalence and Rabies Virus Exposure Risk

**Figure 1:**
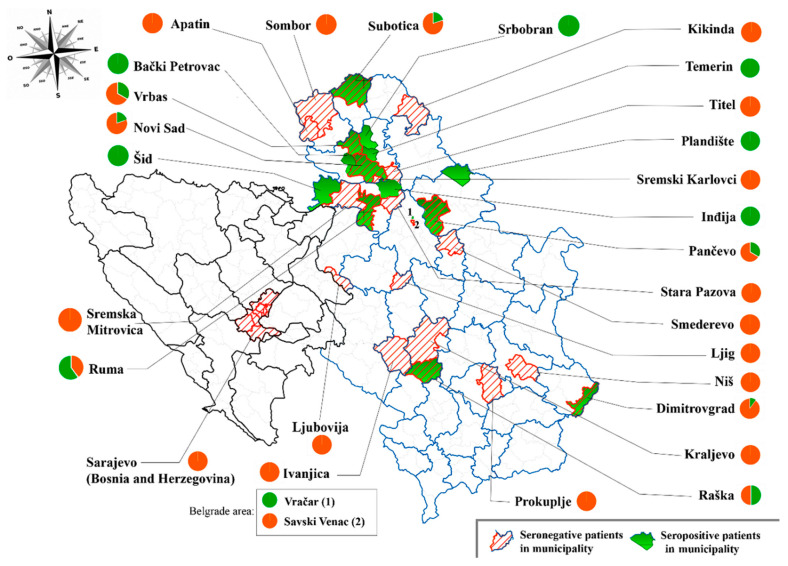
Distribution of seropositive and seronegative samples in study subjects from Serbia (state and district borders marked with blue line) and Bosnia and Herzegovina (state and region borders marked with black line). Municipalities with positive samples are shaded in green, while locations of seronegative samples are crossed with orange lines. Ratio of seropositive and seronegative samples in individuals from one municipality is shown in pie chart near each municipality name (green is for seropositive and orange for seronegative).

When the municipalities of residency of all individuals included in the study were considered, we found a different distribution of *Borrelia* spp. seroprevalence across the Serbian territory. Anti-*Borrelia* IgG were detected in sera from North (17/99, 17.17%; CI, 11.31–27.09), Central (1/11, 9.09%; CI, 0.00–29.26), and South (1/14, 7.14%; CI, 0.00–22.57) Serbia, as well as the Belgrade area (1/2, 50%; CI, 35.22–64.78), while anti-*Borrelia* IgG were not detected in sera samples from Sarajevo in Bosnia and Herzegovina (0/4, 0.0%). Excluding the results from the Belgrade area (due to small sample size *n* = 2), *Borrelia* spp. seroprevalence was higher in Northern Serbia. The local administrative unit level analysis showed that most seropositive patients in Northern Serbia were concentrated in the municipalities Šid, Bački Petrovac, Srbobran, Temerin, Plandište, and Inđija (Figure 1).

When considering *Borrelia* spp. seropositive cases in both PEP and PrEP case groups, no significant differences (χ² = 1.54, *p* = 0.46) were found in seroprevalence between the North (15/69, 21.73%), Central (1/12, 8.33%) and South (2/15, 13.33%) regions of Serbia. As all healthy donor samples were from Northern Serbia, we compared the *Borrelia* spp. seroprevalence among the two case and control groups in Northern Serbia. The difference in *Borrelia* spp. seroprevalence between PEP (6/26, 23.08%; χ² = 3.1, *p* = 0.08) and PrEP (9/43, 20.93%; χ² = 2.8, *p* = 0.09) case groups in the North region was not statistically significant compared with that of the healthy donors control group. However, the OR analysis revealed greater odds of association of *Borrelia* spp. seroprevalence with the rabies virus exposure in PEP (OR = 4.2; CI, 0.88–22.00, *p* = 0.08) and PrEP (OR = 3.7; CI, 0.82–18.00, *p* = 0.09) compared with the healthy donors control group.

### 3.3. Association between Borrelia spp. Seroprevalence and Rabies Virus Exposure Risk According to Age and Gender

**Figure 2:**
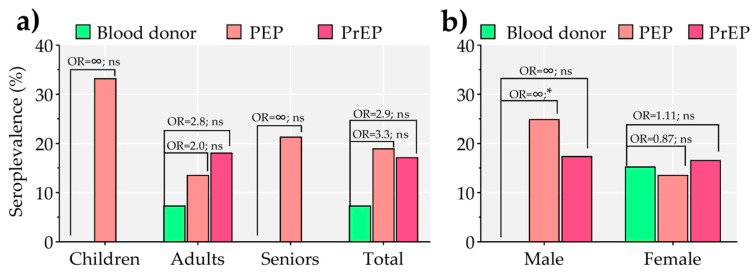
Seroprevalence of anti-*Borrelia* IgG among individuals of the proposed case-control study groups. The case groups PEP (“post-exposure protocol” group) and PrEP (“pre-exposure protocol” group) at risk of rabies virus exposure are compared with a control group (unexposed healthy blood donors). (**a**) *Borrelia* spp. seroprevalence and its association with rabies virus exposure were compared between individuals belonging to the age groups Children (children and teenagers), Adults, and Seniors, as well as overall (Total). (**b**) Comparison of *Borrelia* spp. seroprevalence and association with risk of rabies virus exposure among male and female individuals from the different case-control study groups. OR: odds ratio, ∞ (infinite upper limit) when the prevalence in the control group was 0. Asterisks denote statistically significant differences (* *p* < 0.05, ns: non-significant).

Individuals at risk of rabies virus exposure were unequally distributed in the age groups, and the highest seroprevalence was detected in children and teenagers (2/6, 33.33%; CI, 5.90–75.80), followed by seniors (3/17, 17.64%; CI, 4.67–44.19), and adults (13/77, 16.88%; CI, 9.64–27.50). Due to the fact that PrEP requires the existence of a profession-associated risk of rabies virus exposure, no children or teenagers could undergo this type of vaccination protocol; therefore, comparison of *Borrelia* spp. seroprevalence between PEP and PrEP case groups in this age category was not possible in the present study. There was no significant difference (χ² = 0.23, *p* = 0.62) in the *Borrelia* spp. seroprevalence of PEP (3/22, 13.64%) and PrEP (10/45, 18.18%) case groups. No significant differences (Chi-square test *p* = 1) in *Borrelia* spp. seroprevalence were detected in seniors when PEP (3/14, 21.42%) and PrEP (0/3) case groups were compared between them. In the healthy donors control group, we found anti-*Borrelia* IgG only in two individuals from the adult age group (2/27; 7.41%), while no seropositive individuals were found in seniors (0/1) or children and teenagers (0/2) age groups. In the adults, *Borrelia* spp. seroprevalence was associated with rabies virus exposure risk in the PEP (OR = 2.0; CI, 0.37–12.5; χ² = 0.51, *p* = 0.47) and PrEP (OR = 2.8; CI, 0.65–13; χ² = 1.7, *p* = 0.19) case groups, compared with the healthy donors control group (Figure 2a). In addition, compared with the healthy donors control group of the same age groups, rabies virus exposure risk was not found to be significantly associated with *Borrelia* spp. seroprevalence in children and teenagers (Chi-square test *p* = 1) or seniors (Chi-square test *p* = 1), regardless of immunization protocol.

The analysis of gender distribution in enrolled individuals from case groups showed that 15% (6/40; CI, 3.43–26.57) and 20% (12/60; CI, 9.58–30.42) of seropositive samples to anti-*Borrelia* IgG antibodies were women and men, respectively. No significant difference was found in the *Borrelia* spp. seroprevalence between women and men (χ² = 0.29, *p* = 0. 58). For the healthy donor group, all seropositive samples were from women (2/13, 15.38%; CI, 0.00–38.07). The comparison of the relative odds showed that male individuals of the PEP group had more chance of being seropositive to *Borrelia* spp. compared with the healthy donors (OR = ∞, CI: 1.3–∞; χ² = 4.9, *p* = 0.03) (Figure 2b). No significant differences were found between the PrEP and healthy donor groups (OR = ∞, CI, 0.7–∞; χ² = 3.4, *p* = 0.07). No significant association was found between *Borrelia* spp. seropositivity and the exposure to rabies virus risk in females from PEP (0.87, CI, 0.16-5.5; χ² = 0.02, *p* = 0. 89) and PrEP (1.1, CI, 0.2–7; χ² = 0.01, *p* = 0.92) case groups (Figure 2b).

## 4. Discussion

Lyme borreliosis (LB) is the most common TBD in Europe and is the cause of major concern for both public and veterinary health due to its considerable impact on animal health and human life quality. To the best of our knowledge, this is the first study to consider that individuals at risk of rabies virus exposure also have high odds of *Borrelia* infection. The present study revealed an 18% of *Borrelia* spp. seroprevalence in individuals immunized against rabies, which is higher than any value previously reported by other authors in the healthy population from Serbia [14,27]. In addition, the OR analysis showed a strong association between *Borrelia* spp. seroprevalence with rabies virus exposure risk in the PEP and PrEP case groups, which could be due to activities shared by individuals at risk of rabies and LB, including extensive contact with animals and exposure to tick bites, as reported by most of the study individuals. The lack of statistically significant differences in the comparisons of *Borrelia* spp. seroprevalence between groups may be associated with one of the main limitations of our study (i.e., the small sample size of case and control groups). This finding is in agreement with previous studies conducted in Europe that have described high exposure risk to *B. burgdorferi* s.l. complex in many occupational groups, including forestry workers, farmers, veterinarians, military recruits, and outdoor workers [28,29,30]. It is worth mentioning that anti-*Borrelia* antibodies are not necessarily associated with clinical symptoms of LB and that it is currently unknown for how long these antibodies last in the bloodstream [31]. Therefore, the use of serological tests alone does not suffice to distinguish past from newly acquired infections [32]. The identification of new risk groups related to TBDs is crucial for the improvement and implementation of surveillance programs aimed at the prevention and control of this group of diseases. 

In the present study, subjects immunized against rabies showed higher *Borrelia* spp. seroprevalence and increased likelihood of exposure to ticks and tick-borne pathogens, compared with all previously tested groups from Serbia [17,33]. When the immunization protocol was considered, it became evident that individuals immunized against rabies via PrEP (17.2%) showed similar seroprevalence to soldiers working in the Belgrade area (17.1%) [17]. This finding is explained by the fact that those professionals are more frequently working outdoors in natural environments, where they can be at risk of tick bites, as well as in close contact with animals, including military dogs, hunting dogs, and animals at the veterinary examination centers [17]. On the other hand, subjects immunized via PEP (19.0%) showed similar seroprevalence as Belgrade park maintenance workers (23.5%) and Slovenian forest workers (23.8%) [33,34]. The reason for such similarity in seroprevalence could be the similar lifestyle of these cohorts since edges of forests and places with transitional vegetation have been identified as high-risk areas for humans to ticks exposure [35]. If we consider that the majority of contact with rabid or suspected rabid animals in Serbia could happen in rural areas or wilderness, there is the possibility that PEP patients spend a considerable amount of time in rural environment activities (e.g., recreation, animal husbandry, agriculture, beekeeping, mushroom picking, among others) and possibly live in rural areas where garden maintenance is a common hobby and can share a similar risk of tick bite as those who are involved in park maintenance activity in cities or who work in a forest environment. Nevertheless, it is necessary to conduct further research to clarify which activities related to high risk of tick bite exposure are present in patients that are immunized against rabies via PEP. 

Unfortunately, there are no data about seroprevalence in the general population from Central and South Serbia, which makes it impossible to compare with subjects immunized against rabies from these regions. In addition, seroprevalence found in this study group from Northern Serbia was similar to seroprevalence in professionals at increased risk of LB in Slovenia (21.73% vs. 23.8%) [34]. Previous studies conducted in high-risk professionals for LB from Belgrade reported that seroprevalence of anti-*Borrelia* IgG antibodies in forest workers, park maintenance workers, and soldiers was 11.76%, 23.5%, and 17.14%, respectively [8,21]. In the present study, only two persons immunized against rabies from Belgrade were included, and one of them was seropositive to *Borrelia* spp. Although this is an interesting result, the small sample size makes it impossible to make a meaningful comparison with previous seroprevalence studies in Belgrade. Observed seroprevalence in a population immunized against rabies suggests possible overlap of *Borrelia* exposure risk that was previously linked only to specific professions. Since the majority of seropositive persons are living in northern parts of Serbia, it should be further investigated whether the shared odds of *Borrelia* and rabies virus exposure are present only to specific regions of the country. In regions where shared odds of *Borrelia* and rabies virus exposure are confirmed, a series of preventive measures related to TBDs can be implemented simultaneously with rabies prophylaxis, including educational and screening programs, as well as recommendation of preventive immunizations against ticks or most common TBDs for which vaccines are available. 

In persons who have been infected with *Borrelia* spp. and have achieved the seroconversion, IgG remain detectable for longer periods and have greater specificity for binding to epitopes compared with IgM. For this reason, we only tested exposure to *Borrelia* spp. by screening anti-*Borrelia* IgG. It is considered that *Borrelia* seroprevalence in a region is dependent upon LB endemicity. Except for few studies, there are no published data on *Borrelia* seroprevalence in the human population from other countries bordering Serbia, including Bulgaria, Bosnia and Herzegovina, Montenegro, North Macedonia, and Albania. Countries neighboring Serbia on the north and west with LB endemic regions are Hungary, Slovenia, and Croatia [27,34,36,37,38]. In endemic regions from Slovenia and Croatia, the reported seroprevalence values of anti-*Borrelia* IgG in healthy persons were between 9.7% and 44% [27,34,36]. On the other hand, a study conducted in a population from a non-endemic region in Croatia reported lower seroprevalence values of *Borrelia* spp. (2.7–4.7%) [27]. Similarly, Hristea et al. (2001) reported an overall 4.3% seroprevalence of anti-*Borrelia* IgG antibodies in a healthy population of voluntary blood donors from Romania, which is bordering Serbia on the east [38]. In Serbia, the presence of anti-*Borrelia* IgG has been previously determined in a healthy population from South Bačka District (3.22%) in Northern Serbia and in Belgrade city (2.9–8.57%) [17,33,39]. The variation in *Borrelia* spp. seroprevalence rates in Belgrade could be likely attributed to several factors, including the demography of the sampled population (i.e., the type and number of samples analyzed), the extent of tick infestations, and the sensitivity of diagnostic assays employed [40]. Other studies reported 9.7% and 4.3% *Borrelia* spp. seroprevalence in the Slovenian general population [19] and in blood donors in Romania, respectively [23]. 

## 5. Conclusions

The results of the present study strongly suggest that individuals at risk of rabies virus exposure also have a high likelihood of exposure to tick bites and *Borrelia* infection in Serbia. Although the small sample size and unequal distribution of individuals in both case and control groups are a main limitation of our study, the obtained results suggest that *Borrelia* spp. infection could be a newly recognized occupational hazard in Serbia for professionals working in rabies-risky areas. Individuals following either the PrEP or PEP prophylaxis protocols are good cohorts when considering shared odds of rabies virus and *Borrelia* exposure. The higher distribution of *Borrelia* seroprevalence in several municipalities from Northern Serbia suggests that risk of rabies virus exposure is highly associated with *Borrelia* infection in Northern Serbia. The data obtained in this study indicate that further research is needed to increase the sample size and extend the observations of this study to other TBDs and regions of Serbia. These findings should be considered by physicians and policy-makers to guide risk assessment and public health policies for TBDs at the population level.

## Figures and Tables

**Table 1:** *Borrelia* seroprevalence in individuals at risk of rabies and healthy blood donors.

Groups	Rabies Virus Exposure Risk	Anti-*Borrelia* IgG Detection		*Borrelia* spp. Seroprevalence (%)	CI (95%)
		Negative	Positive		
Healthy donors	No	28	2	6.67	1.16–23.51
“Post-exposure protocol” (PEP)	Yes	34	8	19.05	9.14–34.63
“Pre-exposure protocol” (PrEP)	Yes	48	10	17.24	9.00–29.88

## Supplementary Materials

The following are available online at https://www.mdpi.com/article/10.3390/pathogens10040399/s1, Table S1: Demographic data of individuals vaccinated against rabies following the “post-exposure protocol” (PEP), Table S2: Demographic data of individuals vaccinated against rabies following the “pre-exposure protocol” (PrEP), Table S3: Demographic data of healthy blood donors included in the study.
